# Ce-MOF with Intrinsic Haloperoxidase-Like Activity for Ratiometric Colorimetric Detection of Hydrogen Peroxide

**DOI:** 10.3390/bios11070204

**Published:** 2021-06-23

**Authors:** Yanyan Cheng, Ling Liang, Fanggui Ye, Shulin Zhao

**Affiliations:** State Key Laboratory for the Chemistry and Molecular Engineering of Medicinal Resources, School of Chemistry and Pharmaceutical Sciences, Guangxi Normal University, Guilin 541004, China; 18380539932@163.com (Y.C.); lingliang5200@163.com (L.L.); zhaoshulin001@163.com (S.Z.)

**Keywords:** nanozyme, Ce-MOF, haloperoxidase-like activity, ratiometric colorimetric, hydrogen peroxide

## Abstract

Metal–organic framework (MOF) nanozymes, as emerging members of the nanozymes, have received more and more attention due to their composition and structural characteristics. In this work, we report that mixed-valence state Ce-MOF (MVCM) has intrinsic haloperoxidase-mimicking activity. MVCM was synthesized by partial oxidation method using Ce-MOF as a precursor. In the presence of H_2_O_2_ and Br^−^, MVCM can catalyze oxidative bromination of chromogenic substrate phenol red (PR) to produce the blue product bromophenol blue (Br_4_PR), showing good haloperoxidase-like activity. Because of the special chromogenic substrate, we constructed a ratiometric colorimetric-sensing platform by detecting the absorbance of the MVCM-(PR, Br^−^) system at wavelengths of 590 and 430, for quantifying H_2_O_2_, where the detection limit of the H_2_O_2_ is 3.25 μM. In addition, the haloperoxidase-mimicking mechanism of the MVCM is proposed. Moreover, through enzyme kinetics monitoring, the *K_m_* (H_2_O_2_ and NH_4_Br) of the MVCM is lower than that of cerium oxide nanomaterials, indicating that the MVCM has a stronger binding affinity for H_2_O_2_ and NH_4_Br than other materials. This work provides more application prospects for the development of nanozymes in the field of biosensors in the future.

## 1. Introduction

Nanozymes, as a new type of natural enzyme mimics, have been intensively studied for decades [1]. Compared with natural enzymes, nanozymes have the advantages of diverse structures, good stability in extreme environmental conditions, good repeatability, great recyclability, low cost, and so on [2]. Because of their unique properties, nanozymes have received widespread attention in the field of biosensing in recent years. According to previous reports, applications based on nanozyme sensing mainly contain electrochemical sensing [3], chemiluminescence sensing [4], surface-enhanced Raman scattering (SERS) sensing [5], fluorescence sensing [6], and colorimetric sensing [7]. Among these biosensor methods, sensing methods based on fluorescence and colorimetry have received extensive attention because of their convenience, cheapness, and high sensitivity [8]. Moreover, ratiometric detection is considered to be an ideal method to eliminate most of the interferences [9]. Among the fluorescence sensing methods, the ratiometric fluorescence method has been extensively used in biosensing on account of its advantages of high sensitivity, less interference, and low background [10]. For example, Lin et al. [11] established a ratiometric fluorescent probe based on graphene quantum dots (GQDs) and *o*-phenylenediamine (OPD) to detect the activity of acetylcholinesterase through the catalytic oxidation of OPD by MnO_2_ nanosheets and the fluorescence quenching of GQDs. Su’s team [12] developed a ratiometric fluorescence system to detect bleomycin based on nitrogen-doped graphene quantum dots@gold nanoclusters assembly. Additionally, the experimental phenomenon can be identified by the naked eye, so as to achieve the purpose of visual detection, which is an attractive feature of the colorimetric sensing [13]. Yang et al. [14] reported a two-dimensional, Co_3_O_4_-stabilizing Rh nanocomposite (2D Co_3_O_4_@Rh NC), which has synergistically enhanced the oxidase-like activity between 2D Co_3_O_4_ NS and Rh NPs. The resulting 2D Co_3_O_4_@Rh NC-TMB system can react with urea or *p*-aminophenol with distinguishable color changes, so a ratiometric colorimetric method was established to detect urea and *p*-aminophenol. Although there are many reports on the use of ratiometric fluorescence in biosensing, the application of ratiometric colorimetry is rare. Hence, it is very worthwhile to design a ratiometric colorimetric method for biosensing.

Vanadium haloperoxidases are generally secreted by the algae *Corallina officinalis* and *Delisea pulchra* [15]. Haloperoxidase is a special peroxidase, which can mediate H_2_O_2_ to oxidize halide X^−^ to OX^−^ [16]. Since the chromogenic substrate is phenol red and the brominated product is bromophenol blue, both of which have obvious color changes, the haloperoxidase mimics are particularly suitable for ratiometric colorimetric sensing. So far, V_2_O_5_ nanoparticles, CeO_2−x_ nanorods, and CuO nanoparticles have been found to have haloperoxidase-mimicking activity. For example, Tremel’s group [17] reported that CeO_2−x_ nanorods show haloperoxidase-mimicking activity and the catalytic reaction experiments show mixed valence to play an important role. Compared with CeO_2−x_ nanorods, bulk ceria showed little haloperoxidase-mimicking activity, indicating that the catalytic activity is correlated with the surface area of the nanoscale CeO_2−x_. However, the existing haloperoxidase-mimicking activity is low, and its analytical application is relatively small. As we all know, the activity of the nanozyme is relevant to its shape, size, surface modification, valence, and composition, etc. [18]. Although some reported nanozymes show similar catalytic activity to that of natural enzymes, their intrinsic catalytic activity still has great room for improvement because of fewer exposed active sites, lack of multi-level structure, and their own aggregation. Many nanozymes only involve surface atoms in enzyme-like catalysis, while a large number of internal atoms are either inert or may cause unwanted side reactions, such as many metal oxide nanoparticle-based nanozymes [19].

Different from metal oxide nanoparticle-based nanozymes, metal–organic frameworks (MOFs), as a new type of porous solid material, has well-defined coordination networks, mesoporous structure, high surface area, and adjustable porosity [20]. MOFs also have the advantages of high density and uniformly dispersed active sites. Their porous structure and multi-channels can promote the entry of small molecule substrates and make full contact with the active sites, which are also conducive to the transportation and diffusion of products [21]. Consequently, MOFs are considered to be an ideal material for nanozymes [22]. For example, Jiang’s group [23] reported that MIL-53(Fe) exhibited peroxidase-mimicking activity. It can catalyze the oxidation of OPD, 1,2,3-trihydroxybenzene (THB) and 3,3′,5,5′-tetramethylbenzidine (TMB) in the presence of H_2_O_2_, and its activity is higher than other nanomaterial-based peroxidase mimics. Up to now, several MOF-based nanozymes (MOFzymes) have been reported, but none of them exhibited haloperoxidase-like activity. Hence, it is of great importance to expand the types of MOF-based nanozymes.

**Figure 1:**
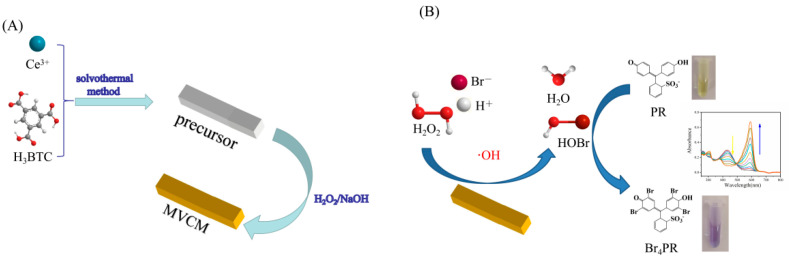
Schematic illustration of (**A**) the synthesis and (**B**) application of the MVCM nanozyme in ratiometric colorimetric detection of H_2_O_2_.

Inspired by CeO_2−x_ nanorod haloperoxidase-mimicking activity, we synthesized a mixed-valence state Ce-based MOF (MVCM) and investigated its haloperoxidase-mimicking activity. In this work, we demonstrate that MVCM possesses an intrinsic haloperoxidase-mimicking activity by catalyzing the bromination of the organic signaling compounds. The reaction principle is that in the presence of H_2_O_2_ and Br^−^, MVCM catalyzes the oxidative bromination of PR, accompanied by a color change; that is, the color of the solution changes from yellow to blue. Due to the bathochromic shift of the absorption peak caused by the oxidative bromination of phenol red to bromophenol blue, a ratiometric colorimetric sensor for detecting H_2_O_2_ was designed (Figure 1). The present work brings new insight into colorimetric sensors and provides a novel, low-cost method for the visual detection of H_2_O_2_, which makes MVCM have a broad application prospect in biomedical analysis and other related fields.

## 2. Experiments

### 2.1. Reagents and Materials

Cerium nitrate hexahydrate (Ce(NO_3_)_3_•6H_2_O), anhydrous ethanol, and sodium hydroxide (NaOH) were acquired from Sinopharm Chemical Reagent Co., Ltd. (Shanghai, China). Phenol red (PR), ammonium bromide (NH_4_Br), potassium bromide (KBr), sodium bromide (NaBr), 1,3,5-benzenetricarboxylic acid (H_3_BTC), acetic acid (AcOH), H_2_O_2_ (30 wt%), sodium acetate (NaOAc), ferric chloride hexahydrate (FeCl_3_•6H_2_O), copper sulfate (CuSO_4_), sodium nitrate (NaNO_3_), sodium chloride (NaCl), magnesium chloride (MgCl_2_), glucose(Glu), lactose (Lac), fructose (Fru), phenylalanine (Phe), mercuric chloride(HgCl_2_), potassium chloride (KCl), sodium sulfate (Na_2_SO_4_), and ammonium oxalate ((NH_4_)_2_C_2_O_4_) were purchased from Beijing HWRK Chem Co., Ltd. (Beijing, China). Celestine blue (Mordant Blue 14, 80%) was obtained from Aladdin (Shanghai, China). All chemicals were analytical reagent grade and used without further purification.

### 2.2. Apparatus

Powder X-ray diffraction (XRD) patterns were recorded with a D/max 2550 VB/PC diffractometer (Rigaku, Japan) using Cu Kα radiation (λ = 0.15418 nm) over a 2θ range of 3–50°. The scanning electron microscopy (SEM) was performed on a FEI Quanta 400 FEG (America FEI). X-ray photoelectron spectroscopy (XPS) data were obtained with a Thermo ESCALAB 250XI electron spectrometer (Thermo, America) using 150 W Al Kα radiation. The Fourier transformed infrared spectroscopy (FTIR) was recorded on a Nicolet iS50 FT-IR spectrophotometer. Ultraviolet-visible (UV-vis) absorbance spectra were measured with a Cary 60 spectrophotometer (Agilent, USA). Electron spin resonance (ESR) was performed on an A300-10/12 Germany Bruker. Matrix-assisted laser desorption/ionization–time-of-flight mass spectrometry (MALDI-TOF MS) analyses were recorded on a Bruker Daltonics (Germany).

### 2.3. Synthesis of MVCM

The MVCM was prepared using the reported partial oxidation method with some modifications [24]. First, a Ce-MOF was prepared by a simple low temperature solvothermal method. Briefly, 8.68 g Ce(NO_3_)_3_•6H_2_O was mixed with 4.2 g H_3_BTC in water/ethanol solution (*v*/*v* = 1:1) and then stirred for 1 h at 60 °C. After centrifuging and washing several times with water and ethanol, the obtained Ce-MOF was dried overnight at 60 °C. Then, the MVCM was prepared by adding 172 μL of a mixture containing NaOH (9.5 mL, 2.5 M) and H_2_O_2_ (0.5 mL, 30 wt%) into the Ce-MOF suspension (40 mg, 8.0 mL). After shaking for 2 min, the yellow solid was centrifuged and washed until the supernatant pH became neutral. After drying overnight at 60 °C, the MVCM was obtained.

### 2.4. Catalytic Activity

Typically, 20 μg/mL of MVCM were added into 2 mL acetic acid buffer (1 mM, pH 4.5) containing 11.25 μM phenol red, 30 mM ammonium bromide, and 200 μM H_2_O_2_. After incubation for 30 min, the absorption spectra of the mixture were determined. For comparison, the absorption spectra of five different systems were measured. In the optimization experiments, the reaction conditions (pH, temperature, incubation time, the concentrations of MVCM, PR and NH_4_Br) and the bromine source were studied.

### 2.5. Analysis of Active Species

ESR measurement was performed by using 5,5-dimethyl-1-pyrroline-N-oxide (DMPO) as the reactive oxygen species trapping agent. The formation of bromine species was detected by celestine blue (CB). The reaction mixture consisted of MVCM, NH_4_Br, CB, and H_2_O_2_, and the CB was bleached in 400 min.

### 2.6. Kinetic Constant Assay

By measuring the apparent steady-state kinetic parameters of the reaction, the haloperoxidase activity of MVCM was further analyzed. The steady-state kinetic values were monitored in time course mode at 590 nm [17]. The maximum initial velocity ($v_{\max}$) and Michaelis–Menten constant ($K_{m}$) were obtained using the Lineweaver–Burk double reciprocal, according to the following equation [25]:

(1) $$\frac{1}{v}=\frac{K_{m}}{v_{max}\left[S\right]}+\frac{1}{v_{max}}$$

Among them, *v* represents the enzymatic reaction speed, *v*_max_ is the maximum enzymatic reaction speed, [S] is the substrate concentration, and $K_{m}$ is the Michaelis constant. In order to assess the kinetic parameters related to the Br_4_PR concentration, the Lambert–Beer law was used to convert the dA_590nm_/dt value to the equivalent d [Br_4_PR]/dt value, and the extinction coefficient of Br_4_PR(ε_Br4PR_) was confirmed to be 72,200 M^−1^ cm^−1^.

(2) $$\left[{\mathrm{Br}}_{4}\mathrm{PR}\right]=\frac{A_{590\mathrm{nm}}}{d\cdot \varepsilon_{{\mathrm{Br}}_{4}\mathrm{PR}}}$$

(3) $$v=\frac{d\left[{\mathrm{Br}}_{4}\mathrm{PR}\right]}{d_{t}}$$

### 2.7. H_2_O_2_ Detection Using MVCM

A distinguishable ratiometric colorimetric method was established to detect hydrogen peroxide. Firstly, 50 μM PR (450 μL), 100 mM NH_4_Br (600 μL), 1 mg/mL MVCM (40 μL), and H_2_O_2_ standard solutions with different concentrations were kept at 37 °C for 50 min, the absorbance at 590 nm and 430 nm was measured by a UV-vis spectrophotometer, and the color changes were compared. In the presence of H_2_O_2_ and Br^−^, MVCM catalyzes the oxidative bromination reaction of PR, turning the solution from yellow to blue. The absorption peak at 430 nm belongs to phenol red, and the absorption peak at 590 nm belongs to bromophenol blue. Through the MALDI-TOF MS analysis of the samples before and after the reaction, it was proved that the product is bromophenol blue. The relationship between A_590nm_/A_430nm_ and the different concentrations of H_2_O_2_ was explored. Therefore, a distinguishable ratiometric colorimetric sensing method was developed, which can be used for H_2_O_2_ detection.

### 2.8. The Analysis of Real Samples

The applicability of the MVCM-PR H_2_O_2_ detection system was studied using the standard addition method. Commercial disinfectants (AL), tap water, milk, and contact lens solutions were chosen as the actual samples. Before testing, the milk was centrifuged at 12,000 rpm to remove the organic content. Then the milk supernatant as well as contact lens solution was diluted 10-fold [26]. Under optimized experimental conditions, the mixture was incubated at 37 °C for 50 min. The absorption peaks of the supernatant at 590 nm and 430 nm were measured.

## 3. Results and Discussion

### 3.1. Characterization of MVCM

**Figure 2:**
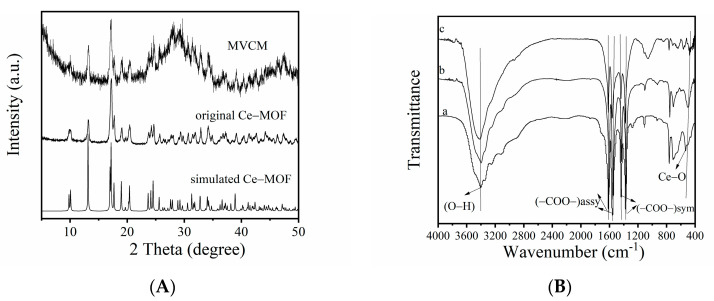

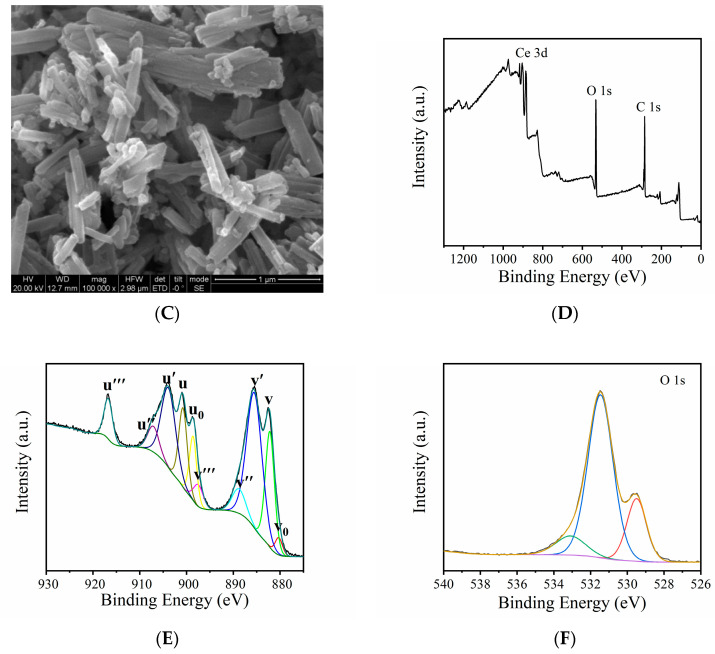
(**A**) XRD patterns of the MVCM and the original Ce-MOF. (**B**) The FTIR spectra of the original Ce-MOF (**a**), MVCM before catalytic reaction (**b**), and after (**c**). (**C**) SEM image of the prepared MVCM. (**D**) XPS survey spectra of MVCM, displaying the Ce 3d XPS spectra (**E**) and O 1s (**F**) of the MVCM.

The crystal structure of the synthesized materials was analyzed by powder XRD (Figure 2A). In Figure 2A, by comparing the synthesized MVCM spectrum with the original Ce-MOF spectrum, it was found that both can be consistent with the standard diffraction peak positions reported in the previous literature [27]. The results showed that the crystal structure of MVCM remained after partial oxidation treatment. In addition, MVCM presented the functional groups similar to that of Ce-MOF according to the FT-IR spectrum (Figure 2B). The characteristic peaks appear in the regions 1617–1553 cm^−1^, 1439–1375 cm^−1^, and 528 cm^−1^, belonging to the stretching vibrations *ν*_assy_ (-COO-) and *ν*_sym_ (-COO-) of the carboxylate ions, and the Ce-O stretching vibration, respectively [28]. The obvious signal at around 3400 cm^−1^ is assigned to the -OH bond. The morphology of MVCM and Ce-MOF was characterized by SEM. In Figure 2C, the synthesized MVCM retains the shape of the nanorod, similar to the prepared original Ce-MOF (Figure S1). X-ray photoelectron spectroscopy (XPS) was used to analyze the elemental composition of the synthesized MVCM. In Figure 2D, the survey spectra of the activated MVCM are shown, illustrating the presence of Ce, C, and O. In Figure S2, the high-resolution C 1s spectrum has two peaks at 284.79 eV and 288.58 eV, which are related to the C=C and C=O bonds, respectively, and the value of -COOH is almost the same [29]. Figure 1E exhibits the high-resolution XPS spectra of the Ce 3D of MVCM, indicating the presence of the Ce^3+^/Ce^4+^ mixed-valence states in MVCM. In the figure, the *v*_0_, *v*′, *u*_0_, and *u*′ peaks belong to Ce^3+^, while *v*, *v*″, *v*‴, *u*, *u*″, and *u*‴ are attributed to the Ce^4+^ ions. The peaks at 882.17, 888.79, 897.46, 900.75, 907.09, and 916.72 eV are related to Ce^4+^, and the peaks at 880.26, 885.50, 898.61, and 903.85 eV are related to Ce^3+^ [29]. By calculating their peak area ratio, the ratio of Ce^3+^/Ce^4+^ in MVCM is 2.0:1.53. Consequently, a Ce-MOF with mixed-valence states was successfully prepared. From Figure 1F, there are three BE peaks in the O1s spectra, with binding energies of 529.56, 531.44, and 533.11 eV, respectively, namely, 529.56 eV (lattice oxygen), 531.44 eV (defective or adsorptive oxygen species), and 533.11 eV (hydroxyl water and/or carbonates) [28,29,30,31].

### 3.2. The Intrinsic Haloperoxidase-Like Activity of MVCM

**Figure 3:**
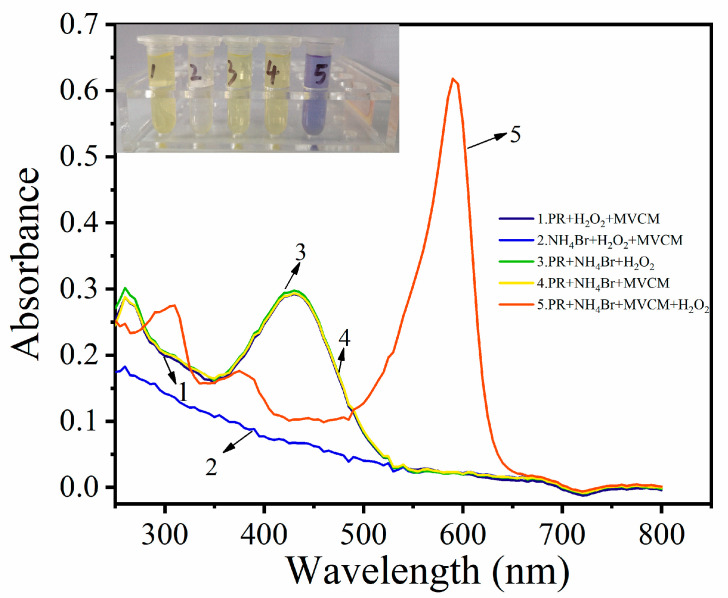
The absorption spectra of the different reaction systems, with the inset showing the corresponding photograph of the different systems.

The haloperoxidase activity of MVCM was proved by a phenol red bromination assay. Using PR as the chromogenic substrate, the haloperoxidase-mimicking activity of MVCM was studied. MVCM catalyzed the bromination reaction of the organic signal compounds (PR and Br^−^) in the presence of H_2_O_2_, turning the solution color into blue, as shown in the Figure 3 inset. In order to explore the haloperoxidase-mimicking activity of MVCM, the absorption spectra of the different reaction systems were recorded. In Figure 3, there is no absorption peak at 590 nm for the PR + MVCM + H_2_O_2_, NH_4_Br + PR + H_2_O_2_, and NH_4_Br + PR + MVCM systems. However, there is a peak at 430 nm, which belongs to PR. In the NH_4_Br + H_2_O_2_ + MVCM system, there is no absorption peak. The PR + NH_4_Br + MVCM + H_2_O_2_ system has an obvious absorption peak at 590 nm, and the absorption peak decreases at 430 nm, which is attributed to the MVCM-catalyzed oxidation bromination of PR by H_2_O_2_ and NH_4_Br to produce bromophenol blue. Only in the fifth system the solution turned blue, indicating that the bromination reaction of the phenol red was caused by MVCM. Through MALDI-TOF analysis, it was proved that MVCM successfully catalyzed the oxidative bromination of phenol red, and the product of the reaction was bromophenol blue. As shown in Figure S3, *m*/*z* 355.161 is the protonated molecular peak of the PR substrate (Figure S3A) and *m*/*z* 670.661 is the base peak of the bromophenol blue proton adduct (Figure S3B).

By comparing the activity of the original Ce-MOF and MVCM, the Ce-MOF catalytic reaction solution had no obvious color change during the whole process of the catalytic reaction. As shown in Figure S4A, Ce-MOF has no absorption peak at 590 nm; on the contrary, MVCM has good haloperoxidase-mimicking activity. This may be due to the mixed-valence states in MVCM. Meanwhile, by comparing the same characteristic quantity of MVCM and CeO_2−x_, the absorption spectra at 430 nm and 590 nm were measured under UV-vis. Under the same conditions, a preliminary comparison between MVCM and CeO_2−x_ shows that the catalytic performance of the former is better than the latter (Figure S4B). This may be due to the high surface area and more exposed active sites of MOF [22].

### 3.3. Active Species Analysis and Catalytic Mechanism

**Figure 4:**
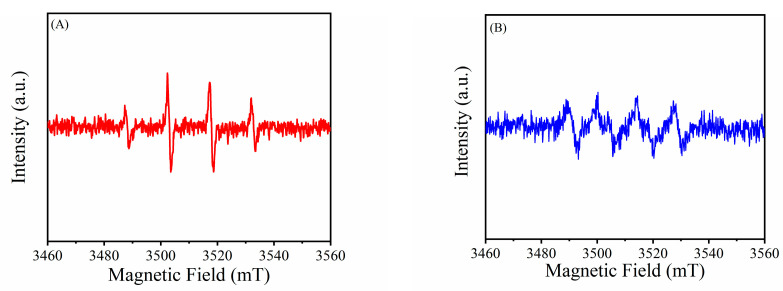
ESR spectrum of the MVCM-H_2_O_2_ system treated with DMPO. (**A**) ESR spectra of DMPO-·OH; (**B**) ESR spectra of DMPO-·O_2_^−^.

The active species generated in the reaction were detected by electron spin resonance (ESR) spectroscopy. 5,5-Dimethyl-1-pyrroline-N-oxide (DMPO) was used as the reactive oxygen sensitive collector. As shown in Figure 4A, the typical OH signals are shown in the MVCM + H_2_O_2_ system, proving that MVCM can catalytically activate H_2_O_2_ to generate OH radicals. At the same time, relatively weak O_2_^−^ radicals were also observed (Figure 4B).

Celestine blue (CB) was used to detect the nature of the intermediate bromine species involved in the PR bromination process. CB is only bleached by oxidizing the halogen species, such as OCl^−^ and OBr^−^, and it is not a substrate of any peroxidase activity and does not react with hydrogen peroxide and superoxide anion [17]. The bleaching of CB at 640 nm within 400 min (Figure S5) showed that the MVCM catalyzed the oxidation of bromide to HOBr/OBr^−^. In Figure S6, only centrifuge tube No. 6 turns pink, while the rest are blue, proving that the CB bleaching is due to the generation of bromine species during the MVCM catalytic reaction rather than the adsorption of the material.

MVCM mimics haloperoxidase to catalyze Br^−^ oxidation to generate HOBr in a mixed solution of NH_4_Br and H_2_O_2_ (Figure S7). Simply described, the H_2_O_2_ molecule can replace the location of the H_2_O and coordinate with the Ce^3+^ site [17]. This condition is unstable, and the H_2_O_2_ molecules tend to dissociate, which will cause Ce^3+^ to be oxidized to Ce^4+^, and formally produce a hydroxyl anion (OH^−^) and a hydroxyl radical (OH) as ligands. The species Br^−^ can be added to an O atom, where one hydroxide anion interacts with the Br radical. However, another non-interacting hydroxide anion is easily protonated, again creating a neutral surface position. Thus, the dissociation of the HOBr product leads to the regeneration of the center of the initial Ce^3+^ site. The generated HOBr can facilitate the bromination reaction of PR to produce Br_4_PR. The above results simply explain the possible catalytic mechanism of MVCM with haloperoxidase-mimicking activity in the mixed solution of NH_4_Br and H_2_O_2_.

### 3.4. Optimal Conditions for H_2_O_2_ Detection

**Figure 5:**
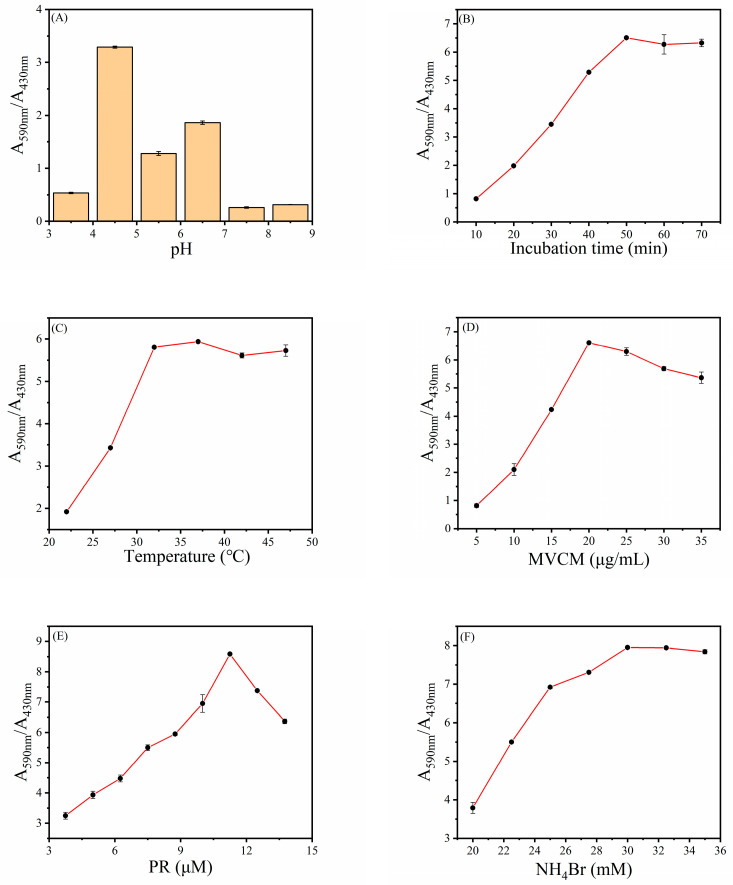
The optimization of (**A**) pH, (**B**) incubation time, (**C**) temperature, and the concentrations of (**D**) MVCM, (**E**) PR, (**F**) NH_4_Br for H_2_O_2_ sensing. Error bars represent the standard deviation of three trials.

The same as natural enzymes, the catalytic activity of MVCM relies on pH, temperature, MVCM concentration, etc. Hence, the optimum conditions of the MVCM catalysis reaction were investigated to ensure the best catalytic activity of the MVCM, such as pH value, incubation time, reaction temperature, and concentration of MVCM, PR, and NH_4_Br. Since the activity of MVCM is closely connected with the pH value, the effect of pH values (3.5, 4.5, 5.5, 6.5, 7.5, and 8.5) on MVCM activity is first studied. Using A_590nm_/A_430nm_ (A_590nm_ and A_430nm_ represent the absorbance of PR and Br_4_PR, respectively) as an index, the best detection conditions for H_2_O_2_ were evaluated. As shown in Figure 5A, when the pH is 4.5, the value of A_590nm_/A_430nm_ reaches the maximum. This is because when the pH is 4.5, the haloperoxidase-mimicking activity of the MVCM is the best. Consequently, we chose 4.5 as the optimal pH value. From Figure 5B one can clearly see that as the incubation time increases, the value of A_590nm_/A_430nm_ gradually increases until it reaches 50 min. The results show that the best reaction time is 50 min. In addition, the incubation temperature of 22–47 °C for this reaction was also studied. Figure 5C shows that with the increase in reaction temperature, the value of A_590nm_/A_430nm_ gradually increases, reaching a plateau at 37 °C. Therefore, in the subsequent experiments, we chose 37 °C as the optimal reaction temperature. Moreover, the material concentration is another important factor that affects the activity of the mimic enzymes. As shown in Figure 5D, along with the increase in MVCM concentration, the value of A_590nm_/A_430nm_ reaches a peak at 20 μg/mL and this concentration is selected as the optimal concentration for the reaction. Additionally, the influence of the concentration of phenol red and ammonium bromide on the reaction system was explored. Figure 5E,F describe the effects of the PR and NH_4_Br concentrations on the intensity of the absorbance ratio. The value of A_590nm_/A_430nm_ reached a peak at a PR concentration of 11.25 μM and reached a plateau at a concentration of 30 mM NH_4_Br. In general, after optimization, the optimal pH value, incubation time, reaction temperature, and concentration of MVCM, PR, and NH_4_Br were 4.5, 50 min, 37 °C, 20 μg/mL, 11.25 μM, and 30 mM, respectively.

In addition, KBr and NaBr served as control samples to evaluate the influence of bromine source. As shown in Figure S8, the absorbance of the solution with different bromine sources is almost the same, indicating that the reaction was independent of bromine sources. Moreover, the effect of different NaOH/H_2_O_2_ volume treatment Ce-MOF on the catalytic activity of the material was explored. Figure S9 explores the effect of four different volumes. Considering the catalytic activity and the maintaining crystalline structure of MVCM, we selected a 172 uL NaOH/H_2_O_2_ volume to treat Ce-MOF.

### 3.5. Steady-State Kinetics Analysis

Using NH_4_Br and H_2_O_2_ as substrates, the steady-state kinetics was used to further study the haloperoxidase-like catalytic mechanism of the MVCM. The kinetic data were collected by changing the concentration of one substrate while keeping the concentration of the other substrate constant. Figure S10A,C show the change in kinetics with the selected substrate concentration when other parameters are constant. The steady-state reaction rates were calculated and applied to the Lineweaver–Burk double reciprocal plot (Figure S10B,D), according to the Michaelis–Menten equation (Equation (1)). The maximum initial velocity (*v*_max_) and Michaelis–Menten constant ($K_{m}$) (Table 1) were obtained using Lineweaver–Burk plots. *K_m_* is generally considered to be an indicator of the affinity of the enzyme to the substrate. The smaller the *K_m_* value, the stronger the affinity of the enzyme to the substrate. Hence, the *K_m_* (H_2_O_2_ and NH_4_Br) of MVCM was lower than that of the cerium oxide nanomaterials, indicating that MVCM had a stronger binding affinity for H_2_O_2_ and NH_4_Br than the other materials (Table S1).

### 3.6. Ratiometric Colorimetric Sensing of H_2_O_2_

**Figure 6:**
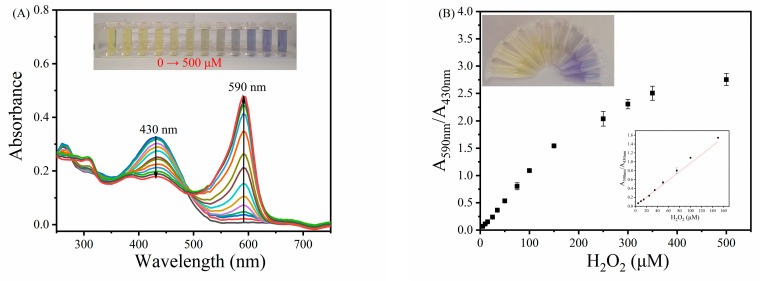
(**A**) The absorption spectra of MVCM-PR system in the presence of different concentrations of H_2_O_2_ (0–500 μM). (**B**) A linear relationship between A_590nm_/A_430nm_ and H_2_O_2_ (*n* = 3). The inset shows the corresponding photographs of the color changes.

Based on the inherent haloperoxidase-mimicking activity of the MVCM, a distinguishable ratiometric colorimetric method for the determination of H_2_O_2_ was established. It is easy to observe from Figure 6A that when the concentration of 0–500 μM H_2_O_2_ is added, the characteristic UV-vis absorption peak of the MVCM-(PR, Br^−^) system at 430 nm gradually decreases, while a new absorption peak appears at 590 nm and gradually increases. It can be directly observed by the naked eye that the color of the MVCM-(PR, Br^−^) system changes from yellow to blue (inset in Figure 6A). There is a good linear relationship between A_590nm_/A_430nm_ and C_H2O2_ in the range of 5.0–150 μM (Figure 6B) under the optimized experimental conditions. The regression equation is A_590nm_/A_430nm_ = 0.00961C_H2O2_ + 0.01084 (R^2^ = 0.9954). According to the definition of the detection limit (detection limit, S/N = 3), the colorimetric detection limit of H_2_O_2_ is 3.25 μM. The analytical performance of the MVCM is comparable to other reports (Table S2).

### 3.7. Selectivity and Applicability of MVCM-Based H_2_O_2_ Detection System

**Figure 7:**
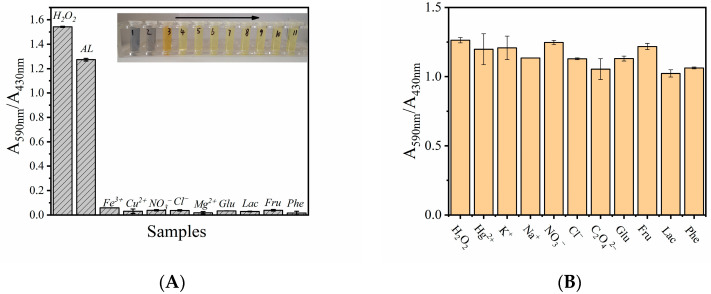
(**A**) Selectivity tests with the 10.0 mM of interferents (Fe^3+^, Cu^2+^, NO_3_^−^, Cl^−^, Mg^2+^, Glu, Lac, Fru, Phe, and diluted AL) of the MVCM-based assay system with 20 μg/mL of the MVCM, 11.25 μM PR, 30 mM NH_4_Br, and an acetic acid buffer (pH = 4.5). Inset: Related color changes. (**B**) Interference tests of the MVCM-based assay system containing the MVCM, acetic acid buffer, PR, NH_4_Br, H_2_O_2_, and interferents (Hg^2+^, K^+^, Na^+^, NO_3_^−^, Cl^−^, C_2_O_4_^2−^ (five times higher than H_2_O_2_), Glu, Fru, Lac, and Phe). The absorbance was monitored at 590 nm and 430 nm after incubating at 37 °C for 50 min. Error bars represent the standard deviation of three trials.

To verify the specificity and feasibility of the H_2_O_2_ detection system based on MVCM-PR, some potential interfering substances were added to the reaction system instead of H_2_O_2_, including Fe^3+^, Cu^2+^, Mg^2+^, NO_3_^−^, Cl^−^, Glu, Lac, Fru, Phe, and commercial disinfectants (AL, about 2.5–3.5% H_2_O_2_, diluted 1000 times). As shown in Figure 7A, although the concentration of these interferers is 10 times higher than that of H_2_O_2_, in the reaction system containing the interferers, the ratio of A_590nm_/A_430nm_ has no obvious change, and the color change is very small. However, the diluted AL system has an obvious blue absorption at 590 nm, and the absorbance decreases at 430 nm. According to the calibration chart shown in the Figure 6B, the concentration of H_2_O_2_ in AL is calculated to be about 0.887 M, which is close to its true concentration (0.816–1.142 M). Therefore, this result shows that the H_2_O_2_ detection system based on MVCM-PR has good specificity and feasibility under some complex conditions.

In order to study the anti-interference property of the H_2_O_2_ detection system based on MVCM-PR, some substances that may appear in the actual analysis were added into the reaction solution, containing Hg^2+^, K^+^, Na^+^, NO_3_^−^, Cl^−^, C_2_O_4_^2−^, Glu, Fru, Lac, and Phe. Although the concentration of these interferers is 10 times higher than that of H_2_O_2_, the sensor system based on MVCM has good anti-interference performance (Figure 7B).

In milk and contact lens solutions, H_2_O_2_ often serve as preservative, stabilizer, and bactericide. Since excess H_2_O_2_ is bad for human health, it is of great significance to detect H_2_O_2_ residues in milk and contact lens solutions. The detection of H_2_O_2_ in actual samples (such as tap water, milk, and contact lens solutions) was evaluated, and the results are shown in Table 2. The recovery rate of the H_2_O_2_ concentration in the above samples by the standard addition method was 92.00–104.40%, and the relative standard deviation (RSD) was less than 2.93%. These data suggest that the MVCM could be used as a probe to detect H_2_O_2_ in real samples such as tap water, milk, contact lens solutions, etc., without being significantly affected by environmental interferences.

## 4. Conclusions

In summary, an MVCM was prepared by partial oxidation with Ce-MOF as the precursor. The haloperoxidase-mimicking activity of the MVCM was proved using the phenol red bromination assay. In addition, we confirmed the active species produced in the reaction catalyzed by the haloperoxidase mimic. The OH radicals and bromine radicals played an important role in the mimic catalytic reaction of the haloperoxidase of the MVCM. Compared with other materials, the MVCM nanozyme material has better substrate affinity. Importantly, in the presence of different concentrations of H_2_O_2_, the resulting MVCM-PR system produces obvious color changes. To study its applicability, a distinguishable ratiometric colorimetric method for detecting H_2_O_2_ was developed. This MVCM nanozyme provides a new form of ratiometric colorimetric sensing.

## Figures and Tables

**Table 1:** Kinetic parameters for the MVCM haloperoxidase mimic.

Material	Substrates	*K_m_* (M)	Vmax (M·s^−1^)
MVCM	H_2_O_2_	1.0 × 10^−4^	4 × 10^−9^
	Br^−^	0.22	1.56 × 10^−9^

**Table 2:** Results of detecting H_2_O_2_ in real samples.

Sample	Original (μM)	Added (μM)	Found (μM)	Recovery (%)	RSD (%) (n = 3)
tap water	N.D.	9.0	8.28	92.00	2.15
	N.D.	80.0	80.94	101.17	2.93
	N.D.	120.0	115.20	96.00	1.30
	N.D.	125.0	130.60	104.40	0.35
milk	N.D.	9.0	8.76	97.33	2.53
	N.D.	80.0	80.34	100.43	1.26
	N.D.	125.0	118.86	95.09	1.15
contact lens solution	N.D.	9.0	8.75	97.22	1.95
	N.D.	80.0	81.07	101.33	2.51
	N.D.	125.0	127.90	102.32	2.75

N.D.: not detected.

## Supplementary Materials

The following are available online at https://www.mdpi.com/article/10.3390/bios11070204/s1, Figure S1: SEM image of the original Ce(III)-MOF; Figure S2: C1s XPS high-resolution spectra of MVCM; Figure S3: MALDI-TOF MS spectra (positive ion modes) of (A) phenol red and (B) bromophenol blue; Figure S4: The absorption spectrum of MVCM and Ce(III)-MOF(A), CeO_2−x_ and MVCM (B) under the same reaction condition; Figure S5: The bleaching of celestine blue (CB) at 640 nm indicates the formation of oxidized bromine species (e.g., OBr^−^); Figure S6: Celestite blue (CB) reacts in different systems. (1. MVCM + NH_4_Br + CB; 2. H_2_O_2_ + NH_4_Br + CB; 3. MVCM + H_2_O_2_ + CB; 4. MVCM + CB; 5. H_2_O_2_ + CB; 6. MVCM + NH_4_Br + H_2_O_2_ + CB); Figure S7: Probable catalytic mechanism of the MVCM as haloperoxidase mimic; Figure S8: Dependence on the bromine source; Figure S9: Ce-MOF was treated with different NaOH/H_2_O_2_ volumes; Figure S10: Steady-state kinetic assays of MVCM. The H_2_O_2_ concentration was varied, the concentration of NH_4_Br and PR was fixed (A); the NH_4_Br concentration was varied, the concentration of H_2_O_2_ and PR was fixed (C); and the double-reciprocal plots of haloperoxidase-like activity of MVCM with a fixed concentration of one substrate relative to varying concentration of the other substrate (B and D); Table S1: A comparison of *K_m_* value for materials-based haloperoxidase mimics; Table S2: Comparison detection limit in different catalyst systems by means of different methods.

## References

[1] Xu X., Luo P., Yang H., Pan S., Liu H., Hu X. (2021). Regulating the enzymatic activities of metal-ATP nanoparticles by metal doping and their application for H_2_O_2_ detection. Sens. Actuators B Chem..

[2] Wu J., Wang X., Wang Q., Luo Z., Li S., Zhu Y., Qin L., Wei H. (2019). Nanomaterials with enzyme-like characteristics (nanozymes): Next-generation artificial enzymes (II). Chem. Soc. Rev..

[3] Li Y., Yu C., Yang B., Liu Z., Xia P., Wang P. (2018). Target-catalyzed hairpin assembly and metal-organic frameworks mediated nonenzymatic co-reaction for multiple signal amplification detection of miR-122 in human serum. Biosens. Bioelectron..

[4] Jia Y., Sun S., Cui X., Wang X., Yang L. (2019). Enzyme-like catalysis of polyoxometalates for chemiluminescence: Application in ultrasensitive detection of H_2_O_2_ and blood glucose. Talanta.

[5] Ma X., Wen S., Xue X., Guo Y., Jin J., Song W., Zhao B. (2018). Controllable Synthesis of SERS-Active Magnetic Metal-Organic Framework-Based Nanocatalysts and Their Application in Photoinduced Enhanced Catalytic Oxidation. ACS Appl. Mater. Interfaces.

[6] Castro R., Soares J., Ribeiro D., Santos J. (2019). Dual-emission ratiometric probe combining carbon dots and CdTe quantum dots for fluorometric and visual determination of H_2_O_2_. Sens. Actuators B Chem..

[7] Sun H., Liu X., Wang X., Han Q., Qi C., Li Y., Wang C., Chen Y., Yang R. (2020). Colorimetric determination of ascorbic acid using a polyallylamine-stabilized IrO_2_/graphene oxide nanozyme as a peroxidase mimic. Microchim. Acta.

[8] Liu H., Ding Y., Yang B., Liu Z., Zhang X., Liu Q. (2018). Iron doped CuSn(OH)_6_ microspheres as a peroxidase-mimicking artificial enzyme for H_2_O_2_ colorimetric detection. ACS Sustain. Chem. Eng..

[9] Wang Z., Yu R., Zeng H., Wang X., Luo S., Li W., Luo X., Yang T. (2019). Nucleic acid-based ratiometric electrochemiluminescent, electrochemical and photoelectrochemical biosensors: A review. Microchim. Acta.

[10] Yang Q., Wang X., Peng H., Arabi M., Li J., Xiong H., Choo J., Chen L. (2020). Ratiometric fluorescence and colorimetry dual-mode assay based on manganese dioxide nanosheets for visual detection of alkaline phosphatase activity. Sens. Actuators B Chem..

[11] Ye M., Lin B., Yu Y., Li H., Wang Y., Zhang L., Cao Y., Guo M. (2020). A ratiometric fluorescence probe based on graphene quantum dots and o-phenylenediamine for highly sensitive detection of acetylcholinesterase activity. Microchim. Acta.

[12] Su D., Wang M., Liu Q., Chen J., Su X. (2019). Dual-emission ratio fluorescence detection of Bleomycin based on nitrogen doped graphene quantum dots@gold nanoclusters assembly. Sens. Actuators B Chem..

[13] Zhan T., Kang J., Li X., Pan L., Li G., Hou W. (2018). NiFe layered double hydroxide nanosheets as an efficiently mimic Enzyme for colorimetric determination of glucose and H_2_O_2_. Sens. Actuators B Chem..

[14] Zhao Q., Zheng X., Xing L., Tang Y., Zhou X., Hu L., Yao W., Yan Z. (2021). 2D Co_3_O_4_ stabilizing Rh nano composites developed for visual sensing bioactive urea and toxic p-aminophenol in practice by synergetic-reinforcing oxidase activity. J. Hazard. Mater..

[15] Sandy M., Carter-Frankin J., Martin J., Butler A. (2011). Vanadiumbromoperoxidase from *Delisea pulchra*: Enzyme-catalyzed formation of bromofuranone and attendant disruption of quorum sensing. Chem. Commun..

[16] Frerichs H., Pütz E., Pfitzner F., Reich T., Gazanis A., Panthöfer M., Jegel O., Heermann R., Tremel W. (2020). Nanocomposite antimicrobials prevent bacterial growth through the enzyme-like activity of Bi-doped cerium dioxide (Ce1-xBixO2-δ). Nanoscale.

[17] Herget K., Hubach P., Pusch S., Deglmann P., Götz H., Gorelik T., Gural’skiy Il’ya A., Pfitzner F., Link T., Schenk S. (2017). Haloperoxidase mimicry by CeO_2−x_ nanorods combats biofouling. Adv. Mater..

[18] Wang Z., Zhang R., Yan X., Fang K. (2020). Structure and activity of nanozymes: Inspirations for de novo design of nanozymes. Mater. Today.

[19] Wang D., Jana D., Zhao Y. (2020). Metal-organic framework derived nanozymes in biomedicine. Acc. Chem. Res..

[20] Lee J., Farha O., Roberts J., Scheidt K., Nguyen S., Hupp J. (2009). Metal-Organic Framework Materials as Catalysts. Chem. Soc. Rev..

[21] Zhang X., Li G., Wu D., Li X., Hu N., Chen J., Chen G., Wu Y. (2019). Recent progress in the design fabrication of metal-organic frameworks-based nanozymes and their applications to sensing and cancer therapy. Biosens. Bioelectron..

[22] Wang F., Chen L., Liu D., Ma W., Dramou P., He H. (2020). Nanozymes based on metal-organic frameworks: Construction and prospects. Trends Anal. Chem..

[23] Ai L., Li L., Zhang C., Fu J., Jiang J. (2013). MIL-53(Fe): A metal-organic framework with intrinsic peroxidase-like catalytic activity for colorimetric biosensing. Chem. Eur. J..

[24] Xiong Y., Chen S., Ye F., Su L., Zhang C., Shen S., Zhao S. (2015). Synthesis of a mixed valence state Ce-MOF as an oxidase mimetic for the colorimetric detection of biothiols. Chem. Commun..

[25] Dutta A., Maji S., Srivastava D., Mondal A., Biswas P., Paul P., Adhikary B. (2012). Synthesis of FeS and FeSe nanoparticles from a single source precursor: A study of their photocatalytic activity, peroxidase-like behavior and electrochemical sensing of H_2_O_2_. ACS Appl. Mater. Interfaces.

[26] Wang Z., Ju P., Zhang Y., Jiang F., Ding H., Sun C. (2020). CoMoO_4_ nanobelts as efficient peroxidase mimics for the colorimetric determination of H_2_O_2_. Microchim. Acta.

[27] Liu K., You H., Jia G., Zheng Y., Huang Y., Song Y., Yang M., Zhang L., Zhang H. (2010). Hierarchically Nanostructured Coordination Polymer: Facile and Rapid Fabrication and Tunable Morphologies. Cryst. Growth Des..

[28] Liu K., You H., Jia G., Zheng Y., Huang Y., Song Y., Yang M., Zhang L., Zhang H. (2018). Hierarchically framework Ce-BTC derivative containing high specific surface area for improving the catalytic activity of CO oxidation reaction. Microporous Mesoporous Mater..

[29] Kouzegaran V., Farhadi K., Forough M., Bahram M., Çetinkol Ö. (2021). Highly-sensitive and fast detection of human telomeric G-quadruplex DNA based on a hemin-conjugated fluorescent metal-organic framework platform. Biosens. Bioelectron..

[30] Deshpande S., Patil S., VNT Kuchibhatla S., Seal S. (2005). Size dependency variation in lattice parameter and valency states in nanocrystalline cerium oxide. Appl. Phys. Lett..

[31] Wang L., Meng F., Li K., Lu F. (2013). Characterization and optical properties of pole-like nano-CeO_2_ synthesized by a facile hydrothermal method. Appl. Surf. Sci..

