# Supplementary material for: Ce-MOF with Intrinsic Haloperoxidase-Like Activity for Ratiometric Colorimetric Detection of Hydrogen Peroxide
Source: Biosensors (Basel). 2021 Jun 23;11(7):204. doi: 10.3390/bios11070204 (PMC8301872; doi:10.3390/bios11070204)
Supplement: Supplementary file 1 [file biosensors-11-00204-s001.zip › biosensors-1220496-supplementary.pdf]

Ce-MOF with intrinsic haloperoxidase-like activity for ratiometric colorimetric detection of hydrogen peroxide

Supplementary Material

Yanyan Cheng, Ling Liang, Fanggui Ye* and Shulin Zhao

State Key Laboratory for the Chemistry and Molecular Engineering of Medicinal Resources, School of Chemistry and Pharmaceutical Sciences Guangxi Normal University, Guilin 541004, P. R. China.

*Correspondence: fgye@mailbox.gxnu.edu.cn; Tel.: +86-773-5856104;

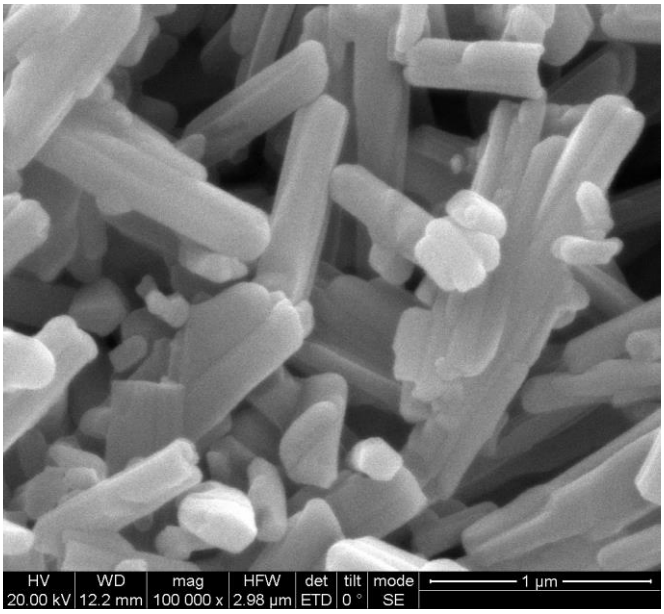

Figure S1 SEM image of the original Ce-MOF.

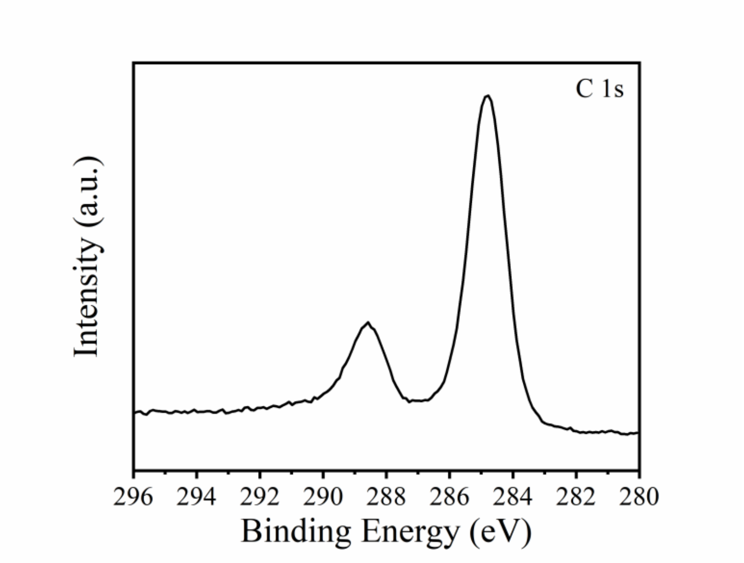

Figure S2 C1s XPS high-resolution spectra of MVCM.

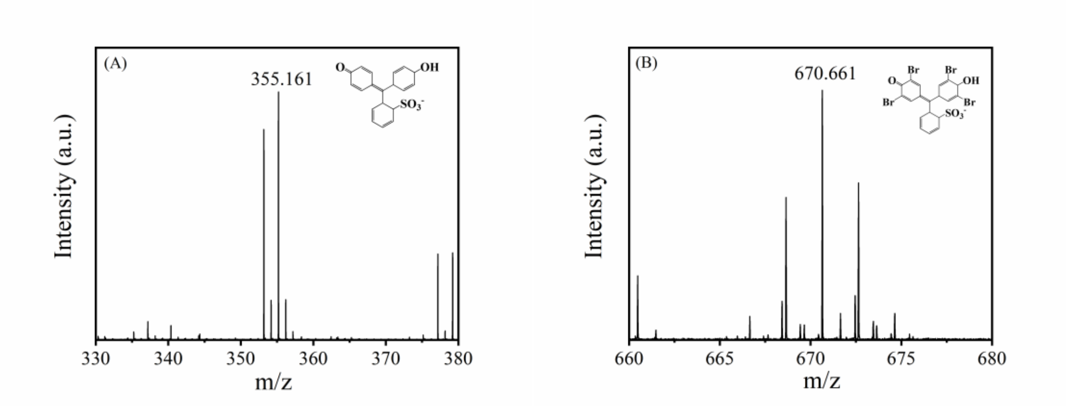

Figure S3 MALDI-TOF MS spectra (positive ion modes) of (A) phenol red and (B) bromophenol blue.

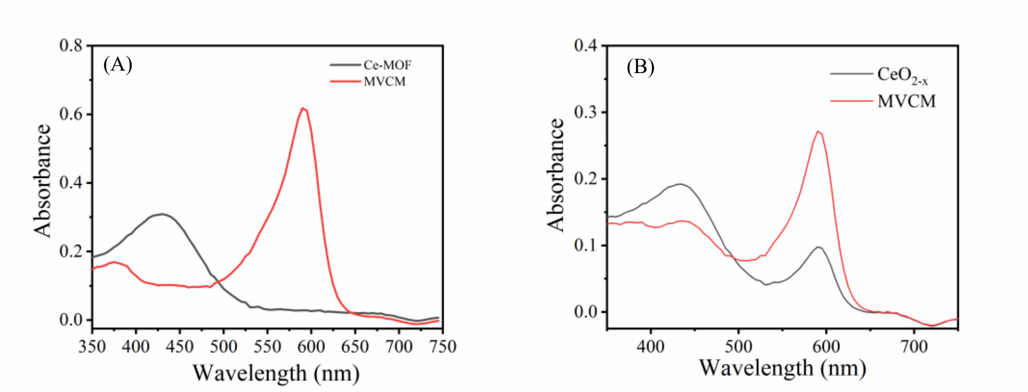

Figure S4 The absorption spectrum of MVCM and original Ce-MOF (A), CeO_{2-x} and MVCM (B) under the same reaction condition.

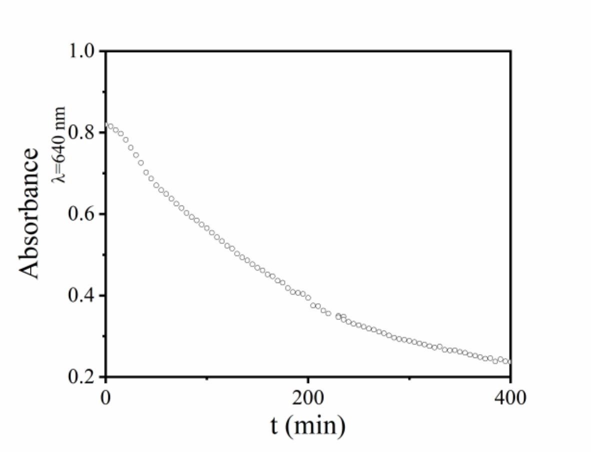

Figure S5 The bleaching of celestine blue (CB) at 640 nm indicates the formation of oxidized bromine species (e.g., OBr^-).

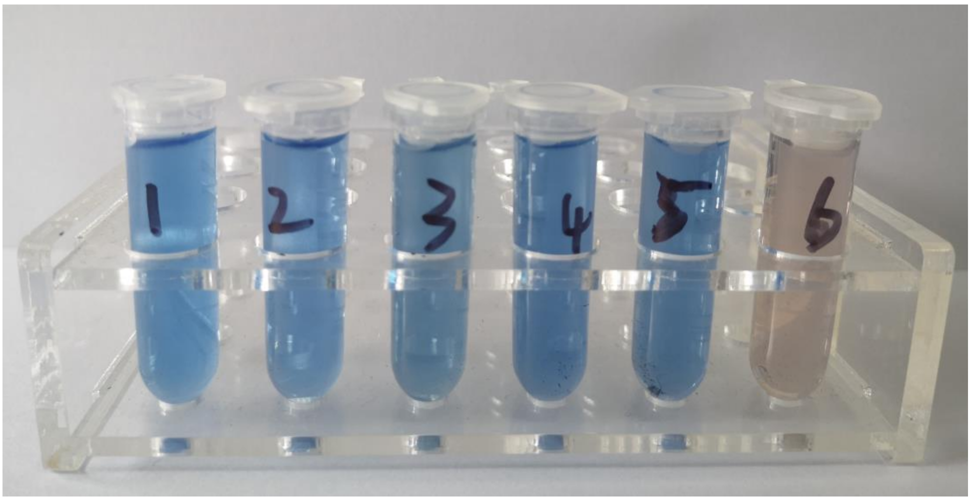

Figure S6 Celestine blue (CB) reacts in different systems. (1. MVCM+NH_4Br+CB; 2.H_2O_2+NH_4Br+CB; 3. MVCM+H_2O_2+CB; 4. MVCM+CB; 5. H_2O_2+CB; 6. MVCM+NH_4Br+H_2O_2+CB).

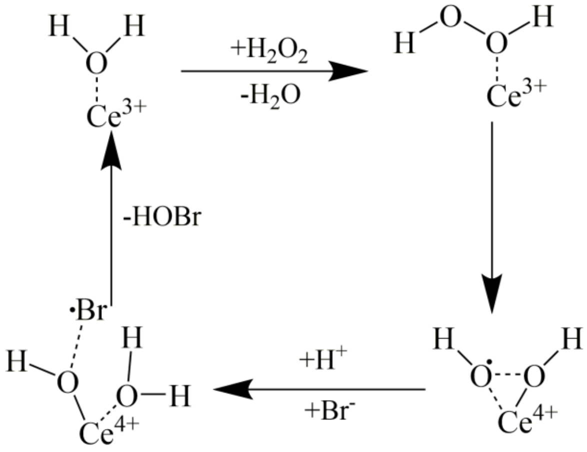

Figure S7 Probable catalytic mechanism of the MVCM as haloperoxidase mimic.

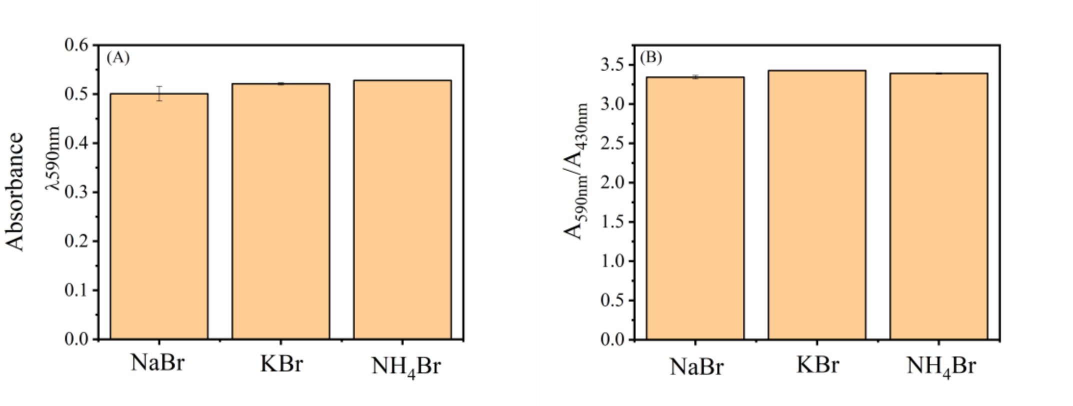

Figure S8 Dependence on the bromine source.

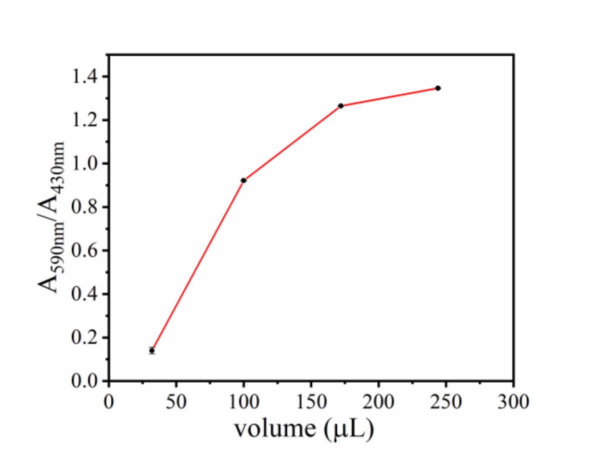

Figure S9 Ce-MOF was treated with different NaOH/H_2O_2 volume.

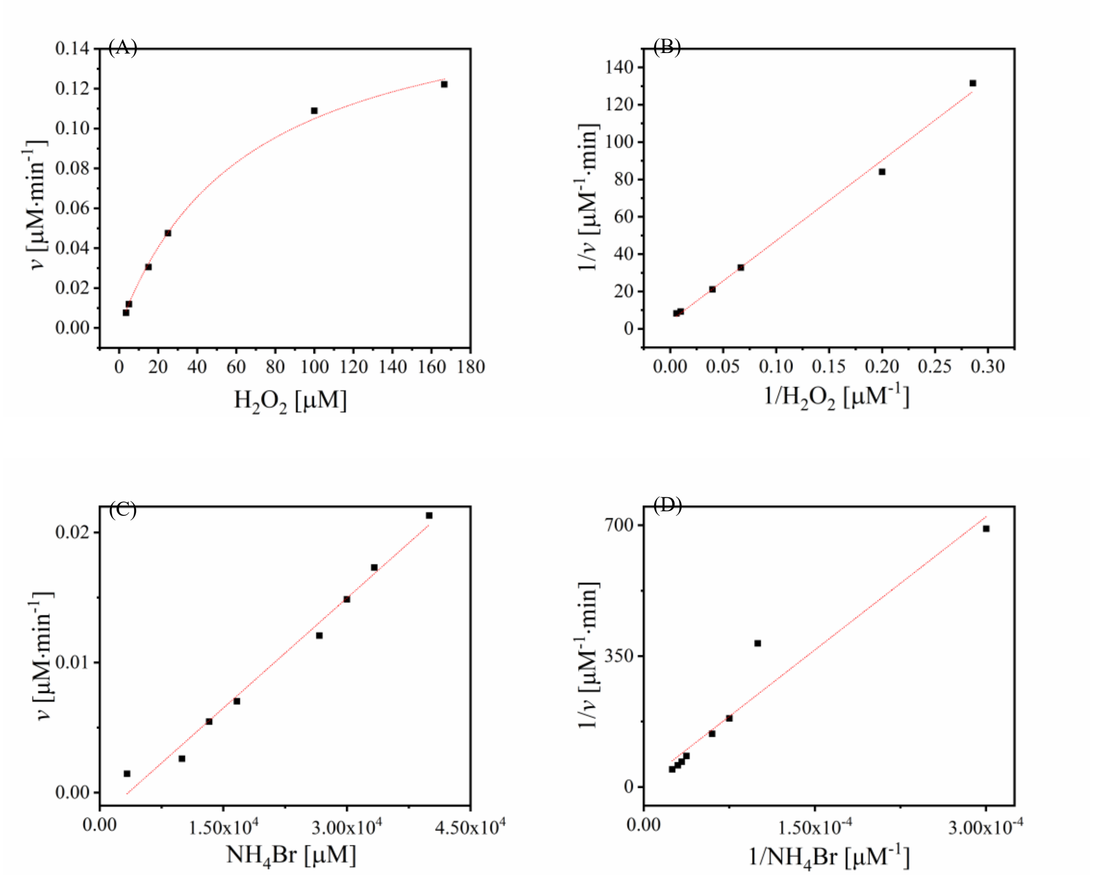

Figure S10 Steady-state kinetic assays of MVCM. The H_2O_2 concentration was varied, the concentration of NH_4Br and PR was fixed (A); the NH_4Br concentration was varied, the concentration of H_2O_2 and PR was fixed (C); and the double-reciprocal plots of haloperoxidase-like activity of MVCM with a fixed concentration of one substrate relative to varying concentration of the other substrate (B and D).

Table S1. A comparison of K_m value for materials-based haloperoxidase mimics.

Materials	Substrate	K_m(M)	Ref.
CeO_2@C	H_2O_2	2.1×10^{-3}	[1]
	Br^-	0.5	
CeO_{2-x} nanorod	H_2O_2	2.6×10^{-4}	[2]
	Br^-	0.35	
MVCM	H_2O_2	1.0×10^{-4}	This work
	Br^-	0.22	

Table S2. Comparison detection limit in different catalyst systems by means of different methods.

Materials	Method	Detection limit (μM)	Ref.
flake-like Cu_2O	Electrochemistry	90.5	[3]
$ZnCrCoO_4$/NCNTs	Electrochemistry	1	[4]
rGO/Ag NPs	Electrochemistry	31.3	[5]
Ce-MOF	Fluorescence	10	[6]
CuO nanoparticles	Fluorescence	0.34	[7][7]
NiFe LDH	Colorimetry	4.4	[8]
Ni^0 nanoparticle	Colorimetry	120	[9]
VS_4	Colorimetry	5	[10]
MVCM	Colorimetry	3.25	This work

Note:

$ZnCrCoO_4$/NCNTs: $ZnCrCoO_4$ and nitrogen-doped carbon nanotubes composite

rGO/Ag NPs: reduced graphene oxide/Ag nanoparticles

NiFe LDH: NiFe layered double hydroxide nanosheets

References

[1] Wang, N.; Li, W.; Ren, Y.; Duan, J.; Zhai, X.; Guan, F.; Wang, L.; Hou, B. Investigating the properties of nano core-shell CeO_2@C as haloperoxidase mimicry catalyst for antifouling applications. Colloids and Surfaces A. 2021, 608, 125592.

[2] Herget, K.; Herget, P.; Pusch, S.; Deglmann, P.; Götz, H.; Gorelik, T.; Gorelik Il'ya, A.; Gorelik, F.; Gorelik, T.; Gorelik, S.; Gorelik, M.; Ksenofontov, V.; Kolb, U.; Opatz, T.; André, R.; Tremel, W. Haloperoxidase Mimicry by CeO_{2-x} Nanorods Combats Biofouling. Adv Mater. 2017, 29, 1603823.

[3] Wen, X.; Long, M.; Tang, A. Flake-like Cu_2O on TiO_2 nanotubes array as an efficient nonenzymatic H_2O_2 biosensor. J Electroanal Chem. 2017, 785, 33-39.

[4] Wu, H.; Xiao, K.; Ooyang, T.;Wang, Z.; Chen, Y.; Li, N.; Liu, Z. Co-Cr mixed spinel oxide nanodots anchored on nitrogen-doped carbon nanotubes as catalytic electrode for hydrogen peroxide sensing. J Colloid Interface Sci. 2021, 585, 605-613.

[5] Liu, S.; Tian, J.; Wang, L.; Sun, X. A method for the production of reduced graphene oxide using benzylamine as a reducing and stabilizing agent and its subsequent decoration with Ag nanoparticles for enzymeless hydrogen peroxide detection. Carbon. 2011, 49, 3158-3164.

[6] Abdelhamid, H.; Sharmoukh, W. Intrinsic catalase-mimicking MOFzyme for sensitive detection of hydrogen peroxide and ferric ions. Microchem J. 2021, 163, 105873.

[7] Hu, A.; Liu, Y.; Deng, H.; Hong, G.; Liu, A.; Xia, X.; Chen, W. Fluorescent hydrogen peroxide sensor based on cupric oxide nanoparticles and its application for glucose and l-lactate detection. Biosens Bioelectron. 2014, 61, 374-378.

[8] Zhan, T.; Kang, J.; Li, X.; Pan, L.; Li, G.; Hou, W. NiFe layered double hydroxide nanosheets as an efficiently mimic Enzyme for colorimetric determination of glucose and H_2O_2. Sens Actuators B

Chem. 2018, 255, 2635-2642.

[9] Zarif, F.; Rauf, S.; Khurshid, S.; Muhammad, N.; Hayat, A.; Rahim, A.; Shah, N.; Yang, C. Effect of pyridinium based ionic liquid on the sensing property of Ni^0 nanoparticle for the colorimetric detection of hydrogen peroxide. J. Mol. Struct. 2020, 219, 128620.

[10] Chao, C.; Wang, Y.; Zhang, D. Peroxidase-like activity of vanadium tetrasulfide submicrospheres and its application to the colorimetric detection of hydrogen peroxide and L-cysteine. Microchim Acta. 2019, 186, 784.
